# Nursing Informatics Competence and Digital Health Readiness Among Chinese Undergraduate Nursing Students: A Convergent Mixed-Methods Study

**DOI:** 10.3390/nursrep16080268

**Published:** 2026-07-31

**Authors:** Yanjing Lv, Minyi Li, Yang Yue, Cynthia Hallensleben, Huohuo Dai, Hongxia Shen

**Affiliations:** 1 School of Nursing, Guangzhou Medical University, Guangzhou 510000, China; lvyanjing@stu.gzhmu.edu.cn (Y.L.); minyi_li@gzhmu.edu.cn (M.L.); 2 Department of Nursing, Guangdong Provincial People’s Hospital, Guangzhou 510000, China; yueyang@gdph.org.cn; 3 Department of Public Health and Primary Care, Leiden University Medical Centre, 2300 RC Leiden, The Netherlands; c.hallensleben@lumc.nl; 4 National eHealth Living Lab, Leiden University Medical Centre, 2300 RC Leiden, The Netherlands

**Keywords:** nursing informatics, digital health, undergraduate nursing students, knowledge, perceptions, skills, mixed-methods study, curriculum development

## Abstract

**Background/Objectives**: Nursing informatics (NI) competence is becoming increasingly essential for nursing students; however, previous research has largely relied on quantitative surveys and provided limited insights into why positive attitudes may not be accompanied by practical competence. This study examined Chinese undergraduate nursing students’ self-assessed NI competence and qualitatively explored their conceptual understanding, perceptions, and experiences related to NI and digital health. **Methods**: A convergent parallel mixed-methods design was employed in Guangzhou, China. Quantitative data were collected from 149 undergraduate nursing students across two nursing schools using the Chinese Self-Assessment of Nursing Informatics Competencies Scale (SANICS), whereas qualitative data were gathered from 23 students at one of the participating schools through six semi-structured focus groups. Quantitative data were analyzed using descriptive statistics and exploratory Spearman correlations, while qualitative data were analyzed thematically. Findings from both strands were integrated during interpretation using a joint display matrix. **Results**: The mean self-assessed NI competence score was 2.78 ± 0.54 (scale 1–5). Among the five SANICS subscales, attitudes toward NI scored highest (4.16 ± 0.76), whereas applied computer skills scored lowest (2.02 ± 0.80), confirming an attitude-competence paradox. Exploratory correlation analyses showed neither year of study nor clinical exposure was significantly associated with the total competence score, although both were negatively associated with attitudes. Three qualitative themes contextualized this paradox: (1) a fragmented conceptual understanding centered primarily on basic functions and tools; (2) positive but cautious perceptions that recognized the value of NI while expressing concerns about humanistic care; and (3) passive skill acquisition, limited hands-on learning, and insufficient awareness of higher-order informatics roles. **Conclusions**: In this two-school sample, students valued NI but reported limited applied and clinically situated competence. The findings suggest that curricula should move beyond generic computer courses and unstructured exposure toward practice-embedded NI education, including electronic health record simulation, digital documentation, data security, and clinically relevant digital skills.

## 1. Introduction

Digital health technologies, broadly defined as technology-enabled tools and systems used across healthcare settings [1], are becoming increasingly embedded in nursing practice. This growing integration has made nursing informatics (NI) competence essential for the delivery of safe, efficient, and high-quality patient care [1,2]. NI integrates nursing science, information science, and computer science to support nursing practice, education, administration, and research [3,4]. Accordingly, undergraduate nursing education should prepare students not merely to recognize digital technologies but to use them safely, effectively, and appropriately within clinical workflows. The American Association of Colleges of Nursing identifies informatics, social media, and emerging technologies as core components of nursing education that shape clinical decision-making and the quality of professional practice [5,6]. NI competence is commonly conceptualized as a multidimensional construct encompassing computer skills, informatics knowledge, and the ability to apply informatics principles in clinical settings [7]. Collectively, these competencies serve as important indicators of nursing students’ preparedness for future digital health practice and technology-enabled care [8].

In China, the National Health Commission has identified NI competence as an important component of nurses’ professional development [9]. Digital health tools can support information integration across nursing services, improve workflow efficiency, and enhance the quality and safety of care [9,10]. Nevertheless, nursing students may encounter difficulties in transferring general digital skills to clinical practice and may overestimate their preparedness for informatics-related tasks [8]. Consequently, positive attitudes toward NI do not necessarily translate into the competence required to use digital technologies safely and effectively in clinical settings. This discrepancy may compromise students’ ability to document patient information accurately, use nursing information systems appropriately, identify technology-related risks, and participate effectively in digital health innovation. From an educational perspective, failure to address this gap may leave future nurses inadequately prepared for technology-enabled healthcare environments, in which informatics competence increasingly shapes care quality, efficiency, and patient safety. Previous studies have similarly reported that nursing students may hold favorable perceptions of NI while demonstrating limited practical competence [1,10]. In this study, we use the term “attitude–competence paradox” as an explanatory concept and interpretive lens for this recurring discrepancy, rather than as a formally established theoretical construct.

Existing evidence suggests that undergraduate nursing students generally report low-to-moderate levels of self-assessed NI competence [1,10]. While quantitative studies have documented the attitude-competence paradox, they cannot explain why it occurs or how educational experiences shape this discrepancy. One plausible explanation is inadequate learning transfer. When informatics is taught primarily as a stand-alone computer-related subject rather than being embedded in nursing-specific scenarios, students may acquire general technical knowledge without developing the ability to apply it in clinical contexts. Understanding this discrepancy requires moving beyond surveys to examine how students actually learn about NI and digital health. Specifically, it is important to understand the educational contexts in which students develop (or fail to develop) applied informatics skills. This study draws on situated learning theory to interpret why informatics education embedded in clinical contexts may be more effective than decontextualized instruction. Situated learning theory proposes that professional competence develops through meaningful participation in authentic practice contexts rather than through decontextualized instruction alone [11]. Therefore, a qualitative exploration of students’ learning experiences is necessary to identify the contextual and educational factors that may contribute to this discrepancy, including passive modes of learning, fragmented curriculum design, and limited opportunities for authentic clinical application. By integrating quantitative assessments of competence and attitudes with qualitative accounts of students’ experiences, mixed-methods research can move beyond determining whether an attitude–competence paradox exists to provide a more comprehensive explanation of how and why it emerges in undergraduate nursing education.

Accordingly, this study advances beyond merely describing competence levels to providing contextual explanations for the attitude-competence paradox through the integration of quantitative and qualitative data. Using a convergent parallel mixed-methods design [12,13], this study aimed to: (1) quantify self-assessed NI competence among Chinese undergraduate nursing students, (2) qualitatively explore their conceptual understanding, perceptions, and learning experiences related to NI and digital health, and (3) integrate quantitative and qualitative findings to elucidate the attitude-competence paradox.

## 2. Materials and Methods

### 2.1. Study Design

A convergent parallel mixed-methods design was used to develop a multidimensional understanding of undergraduate nursing students’ NI competence, perceptions, and learning experiences. This design was selected because it enabled simultaneous examination of the quantitative patterns of NI competence and the qualitative contextual explanations of students’ learning experiences, allowing this study to address both what patterns exist and why they may occur. The quantitative survey and qualitative focus groups were conducted during the same general data-collection period, and neither strand was designed as a follow-up to the results of the other. The two strands were analyzed independently and integrated during interpretation using a joint display matrix.

The purpose of integration was not to establish statistical equivalence or complete convergence between identically composed samples. Instead, integration was used to examine whether the qualitative accounts were consistent with, added contextual depth to, or diverged from the principal patterns identified in the survey.

### 2.2. Study Setting and Participants

This study was conducted at two public undergraduate nursing schools located in Guangzhou, Guangdong Province, China. Both institutions offered Bachelor of Science in Nursing programs within the same urban metropolitan and regional educational context. The participating schools represented urban nursing education settings in China and provided the context for examining undergraduate students’ NI competence and learning experiences. The target population for both study strands comprised students currently enrolled in an undergraduate nursing program.

The same general eligibility criteria were applied to both strands. Students were eligible if they were currently enrolled in a formal undergraduate nursing program and were willing to provide written informed consent. Students on approved academic leave were excluded.

For the quantitative strand, students from all academic years at the two participating schools were eligible for recruitment to describe patterns of self-assessed NI competence across the accessible student population.

For the qualitative strand, purposive sampling was conducted at one of the participating schools to recruit information-rich participants with variation in academic year and prior exposure to nursing information systems. The qualitative component was limited to one school because this institution provided feasible access for in-person focus group discussions during the data-collection period. Fourth-year students were undertaking off-campus clinical placements and were therefore unavailable for the planned face-to-face focus groups; consequently, the qualitative sample comprised students from Years 1–3.

Although qualitative data were collected from only one of the two participating schools, the educational curricula across the two schools were comparable in structure and content (verified through curriculum review). Nevertheless, we recognize that this may constrain the transferability of qualitative findings to the context of the second school. Sample composition differences were considered when interpreting cross-strand integration.

### 2.3. Operational Definitions of Key Concepts

To improve conceptual clarity, the three core concepts investigated in this study (knowledge, perceptions, and skills) were operationally defined prior to data collection. Because this mixed-methods study combined a self-assessment questionnaire with qualitative interviews, these concepts were derived from different sources of evidence rather than a single measurement instrument. Their operational definitions and corresponding measurements are presented in Table 1.

### 2.4. Research Materials

#### 2.4.1. The Demographic and General Questionnaire

A demographic questionnaire collected information such as age, gender, grade, hometown, exposure to information systems/devices, clinical practice and computer course experience. Clinical exposure was assessed by asking participants whether they had experienced several predefined forms of clinical learning, including clinical observation, skills training, and clinical placement. Clinical exposure was measured as the number of exposure types reported, which provides a proxy measure but does not capture the duration or quality of clinical informatics experiences.

#### 2.4.2. Self-Assessment of Nursing Informatics Competence (Quantitative Phase)

Within the operational framework of the present study, the competence-related domains of SANICS were used to represent students’ self-perceived NI skills, whereas the attitudes domain was used to represent perceptions toward NI and digital health. Conceptual knowledge was explored qualitatively through focus group discussions rather than objective testing. To quantitatively measure students’ self-assessed NI competence, participants completed the validated Chinese version of the Self-assessment of Nursing Informatics Competencies Scale (SANICS) [14], originally developed by Yoon et al. [15], which measures perceived competence and attitudes rather than objectively demonstrated informatics performance. It contains 28 items rated on a five-point Likert scale (1 = not competent to 5 = expert), with higher scores indicating higher self-assessed NI competence. Because validated categorical cut-offs for low, moderate, or high competence have not been established for this sample, score levels were interpreted descriptively by reference to the five-point response range. The Chinese SANICS has demonstrated good reliability (Cronbach’s alpha = 0.931) and validity [14]. The items are categorized into five subscales: clinical informatics role (items 1–5), basic computer knowledge and skills (items 6–16), applied computer skills (items 17–20), wireless device skills (items 21–24), and NI attitudes (items 25–28) [14,15]. Internal consistency was reassessed in the present sample using Cronbach’s alpha for the total scale and its five subscales. Table 2 presents the five domains with example items.

#### 2.4.3. Focus Group Discussion Topic Lists (Qualitative Phase)

The interview guide was designed to explore participants’ conceptual knowledge, perceptions, and self-reported learning and application experiences related to NI and digital health. A semi-structured discussion guide was developed based on the previous literature [16,17]. The guide included open-ended questions designed to elicit participants’ understanding of NI and digital health, their views on the importance of these competencies, their perceived readiness for informatics-rich clinical environments, and factors that facilitated or hindered the development of these skills (Additional File S1).

### 2.5. Sample Size

For the quantitative phase, sample size was estimated using an item-to-participant ratio commonly applied in questionnaire-based studies [18]. The SANICS contains 28 items; therefore, the lower bound of 5 participants per item indicated a minimum recommended sample size of 140 participants [18]. The final sample included 149 students, corresponding to approximately 5.3 participants per item. This heuristic approach was considered appropriate for the descriptive and exploratory aims of this study; however, future hypothesis-driven studies involving inferential or predictive modeling should determine sample size using formal a priori power analysis. Future hypothesis-driven studies should conduct formal a priori power analyses using expected effect sizes derived from this exploratory work.

For the qualitative phase, purposive maximum-variation sampling was used within the accessible student population to obtain information-rich accounts of NI learning experiences. Sampling aimed to achieve variation in academic year, previous computer-course experience, exposure to nursing information systems, and clinical-learning experience rather than statistical matching with the quantitative sample. Recruitment continued until thematic sufficiency was reached, defined operationally as the point at which two consecutive focus groups generated no substantively new codes or conceptual insights relevant to the study aim [19,20].

### 2.6. Data Collection

#### 2.6.1. Quantitative Phase

The online SANICS survey was distributed between June and July 2024 to undergraduate nursing students in the two participating schools. A total of 150 questionnaires were distributed, of which 149 valid responses were returned and included in the analysis, yielding an effective response rate of 99.3%. Participation was voluntary, confidential, and anonymous. Written informed consent was obtained before survey completion.

#### 2.6.2. Qualitative Phase

Focus group discussions were conducted in Mandarin Chinese and moderated by a researcher with qualitative research training and experience in conducting semi-structured interviews. To minimize potential power-related influences and encourage open sharing, the moderator had no prior relationship with the participants, held no teaching or administrative responsibilities over them, and did not participate in their academic evaluation. Participants were explicitly reminded that their participation was voluntary and would not affect their grades. To further mitigate potential researcher bias and enhance reflexivity, the moderator utilized a semi-structured interview guide, avoided evaluative responses or leading questions during discussions, and recorded reflexive notes after each session. Discussions were held in a quiet, private classroom, lasted approximately 50–60 min, and were audio-recorded with permission. Recordings were transcribed verbatim in Chinese, checked against the audio files for accuracy, anonymized, and translated into English for reporting. Translations were cross-reviewed by bilingual members of the research team to preserve original meanings and nuances.

### 2.7. Data Analyses

#### 2.7.1. Quantitative Analysis

Data were analyzed using IBM SPSS Statistics version 21.0 (IBM Corp., Armonk, NY, USA). Descriptive statistics, including means, standard deviations, frequencies, and percentages, were used to characterize the sample and summarize the SANICS total and subscale scores. The quantitative analysis was primarily descriptive because the main aim was to characterize patterns of self-assessed NI competence rather than to test prespecified hypotheses or construct a predictive model. In addition, participant distribution across several demographic subgroups was uneven, particularly by academic year and sex, which limited the reliability of extensive subgroup comparisons.

In response to the exploratory potential of the collected educational variables, Spearman’s rank correlation analyses were conducted to examine associations between year of study, prior exposure to nursing information systems, computer course experience, clinical exposure level, and the SANICS total and subscale scores. Year of study and clinical exposure level were treated as ordinal variables. Prior exposure to nursing information systems and computer-course experience were coded as binary variables (0 = no and 1 = yes). Spearman’s rank correlation was selected because it is appropriate for ordinal data, is less sensitive to departures from normality, and permits exploratory assessment of monotonic associations.

Correlation coefficients are reported as Spearman’s rho with two-tailed *p* values. Because these analyses were exploratory and involved multiple comparisons, emphasis was placed on the direction, magnitude, and consistency of associations rather than on isolated *p* values. No adjustment for multiple testing was applied; therefore, statistically significant findings should be interpreted cautiously and considered hypothesis-generating rather than confirmatory. The cross-sectional design also precludes causal interpretation.

#### 2.7.2. Qualitative Analysis

Transcripts were analyzed using Atlas.ti for Windows V.7.5.18 (Scientific Software Development, Berlin, Germany). Inductive thematic analysis followed Braun and Clarke’s six-phase approach [21]. Two researchers independently read the transcripts and generated initial codes. They then compared codes, discussed discrepancies, and refined the coding framework through consensus; any unresolved disagreements were discussed with a third member of the research team. Codes were subsequently grouped into candidate subthemes and themes, which were reviewed iteratively against the original transcripts to ensure accurate representation of participants’ experiences. Data collection and analysis proceeded iteratively, and thematic saturation was considered to have been reached when continuous comparative analysis of the transcripts revealed that no substantially new codes, themes, or perspectives were emerging [19,20]. Rigor and trustworthiness were further supported through peer debriefing, reflexive journaling by the researchers, and detailed documentation of analytic decisions [21,22].

#### 2.7.3. Methods for Integrating Quantitative and Qualitative Findings

The quantitative and qualitative strands were analyzed independently before integration. Integration was conducted at the interpretation stage using a side-by-side joint display matrix, following the mixed-methods integration principles described by Fetters et al. [13]. For each core dimension of students’ informatics readiness—including knowledge (e.g., conceptual understanding), perceptions, and applied competence—the main quantitative patterns were aligned with relevant qualitative themes and illustrative quotations.

The research team iteratively examined whether the qualitative findings were consistent with, expanded upon, or provided contextual explanations for the survey patterns. Because the two samples differed in institutional coverage and academic-year composition, integration was not intended to demonstrate complete convergence between statistically identical populations. Instead, it was used to identify complementary evidence and to develop contextually grounded interpretations of the survey findings. Differences in sample composition were explicitly considered when drawing integrated conclusions.

### 2.8. Ethics Approval and Consent to Participate

This study was conducted in accordance with the Declaration of Helsinki. This study was approved by the Ethics Committee of the University Ethics Committee of Guangzhou Medical University (Reference Code: L202303012). All participants received a detailed explanation of the study’s purpose and procedures. Written informed consent was obtained from all participants prior to data collection. All data were collected anonymously and confidentially, with no personal identifiers used in data processing or publication.

## 3. Results

### 3.1. Quantitative Findings

#### 3.1.1. Participant Demographic Characteristics

A total of 150 undergraduate nursing students from two nursing schools in Guangzhou, Guangdong Province, were invited to participate in the survey. Of these, 149 students completed the survey and provided valid responses, yielding a response rate of 99.3%. The final sample consisted of 16 male students (10.7%) and 133 female students (89.3%), with the majority aged 21–24 years. Detailed demographic characteristics are presented in Table 3.

#### 3.1.2. Self-Assessed Nursing Informatics Competence

The overall mean self-assessed SANICS score was 2.78 ± 0.54, indicating a level slightly below the midpoint of the five-point response scale (Table 4). The highest score was observed for attitudes toward NI (4.16 ± 0.76), whereas the lowest scores were found for applied computer skills (2.02 ± 0.80) and clinical informatics role (2.39 ± 0.74). Notably, the mean attitude score was more than double the mean applied computer skills score, illustrating the attitude–competence paradox identified in this study. The SANICS demonstrated excellent internal consistency in the present sample, with a Cronbach’s alpha of 0.946 for the total scale. Cronbach’s alpha coefficients for the five subscales ranged from 0.906 to 0.943, indicating good to excellent internal consistency across dimensions. The five highest- and five lowest-scoring individual items are presented in Table 5.

#### 3.1.3. Exploratory Spearman Correlation Analyses

Exploratory Spearman’s rank correlation analyses were conducted to examine associations between selected educational variables and the SANICS total and subscale scores. The full correlation coefficients and two-tailed *p* values are presented in Table 6.

Year of study, prior exposure to nursing information systems, computer-course experience, and clinical exposure level were not significantly associated with the SANICS total score. Most associations with the competence-related subscales were also nonsignificant. However, higher year of study and greater clinical exposure were associated with less positive attitudes toward NI, whereas computer-course experience was associated with more positive attitudes but lower clinical informatics role scores.

Greater academic progression or broader clinical exposure did not consistently correspond to higher self-assessed NI competence. The significant associations were concentrated mainly in the attitude domain and varied in direction across educational variables. The negative correlations between year of study or clinical exposure and attitudes may reflect students’ increasing awareness of practical limitations in clinical information systems, less favorable experiences with system usability or implementation, differences in educational experiences across cohorts, or variations in clinical learning environments. However, these interpretations remain tentative because the analyses were exploratory and cross-sectional and involved multiple comparisons and uneven subgroup distributions. Therefore, no causal or developmental inference should be drawn from these associations.

Taken together, these exploratory results suggest that greater academic or clinical exposure did not necessarily correspond to higher self-assessed NI competence. In particular, exposure-related variables were more consistently associated with attitudes than with applied competence domains. Because these analyses were exploratory, involved multiple comparisons, and were based on a cross-sectional design with uneven subgroup distributions, the observed associations should be interpreted cautiously and regarded as hypothesis-generating rather than confirmatory.

### 3.2. Qualitative Findings

#### 3.2.1. Participant Characteristics

Using purposive sampling, 23 undergraduate nursing students from one of the two participating nursing schools were recruited for six focus groups, with three to four participants in each group (Table 7). The qualitative sample included students from Years 1–3 and comprised 20 female and 3 male students aged 19–21 years. All participants had completed a computer-related course, 11 reported previous exposure to nursing information systems, and 22 reported some form of clinical-learning experience through observation, skills training, or placement.

Participants were selected to provide variation in academic year and relevant educational and digital-health exposure within the accessible population Years 1–3 population. Fourth-year students were unavailable because they were undertaking off-campus clinical placements during the data-collection period. Therefore, the qualitative findings therefore primarily reflect the experiences of pre-graduation students in Years 1–3 and should not be interpreted as fully representing the transition-to-practice experiences of fourth-year students.

#### 3.2.2. Thematic Analysis Overview

Three interrelated themes and their corresponding subthemes were derived from the analysis (Table 8 and Table 9). Theme 1, Knowledge of NI and Digital Health, reflected fragmented and predominantly functional conceptual understanding, with students focusing mainly on basic definitions and commonly used digital tools. Theme 2, Perceptions of NI and Digital Health, captured students’ generally positive views regarding the efficiency and clinical value of NI, alongside cautious concerns about its potential implications for humanistic care. Theme 3, Skills and Clinical Role Cognition, reflected predominantly passive skill-acquisition pathways, limited opportunities for clinically relevant hands-on practice, and emerging but uneven awareness of higher-order nursing informatics roles. Selected illustrative quotations are presented in Table 8 and Table 9, with expanded original quotations provided in Additional File S2. The distinction among raw quotations, initial codes, subthemes, and analytical themes was maintained throughout the coding and analytic process.

Theme 1: Knowledge of NI and Digital Health
Conceptual understanding

Students’ conceptual understanding of NI was fragmented and mostly functional. Most defined NI as the use of computers, information analysis, or digital tools to improve patient care [Q1], while fewer described its interdisciplinary nature or links with nursing science, information science, and computer science [Q2]. Their knowledge was also shaped by national digital health policies and clinical exposure, including references to Internet + Nursing and hospital information systems [Q3–Q7]. Students could identify important principles such as privacy protection, information accuracy, and patient-centered use of technology, but they rarely connected these principles to advanced system design, data governance, or clinical decision-support functions.

Application tools

Students’ knowledge of clinical digital tools was related to basic equipment and surface-level familiarity. Most could name common tools such as electronic medical records and describe their general function in nursing work [Q8]. Some students demonstrated awareness of tools used in data collection and nursing work assessment [Q9–Q10]. However, their descriptions focused mainly on tool recognition rather than on workflow integration, data quality, interoperability, or safe clinical decision-making.

Theme 2: Perceptions of NI and Digital Health
Recognition of application value

Students acknowledged the benefits of NI in clinical practice. The most commonly cited advantage was improved work efficiency, with students noting that digital health saves time and effort, thereby reducing medical staff workload [Q11]. Students also recognized broader benefits, including enhanced efficiency for both doctors and patients, as well as increased time for nurses to dedicate to direct patient care [Q12].

Awareness of limitations

A small number of students expressed concerns about the potential limitations of NI. They emphasized that while technology can support physical care, nursing must also address patients’ psychological and humanistic needs, an area they felt requires further strengthening in the context of digital health [Q13].

Theme 3: Skills and Clinical Role Cognition
Skill acquisition pathways

Students reported limited access to NI skill development, relying primarily on on-campus courses and off-campus clinical practice. Many acknowledged that their understanding remained superficial despite exposure to relevant courses and lectures [Q14]. One student noted that the programming language taught in class was impractical, while a more accessible language was only briefly introduced without sufficient depth [Q15]. Few students engaged in proactive self-directed learning, with most passively receiving information through formal curricula.

Basic clinical role cognition

Students identified several operational and intermediate roles that nurses play in NI. Participants described nurses as users of computerized systems and providers of clinical feedback, including identifying inappropriate system design and suggesting improvements based on clinical needs [Q16]. They also recognized nurses’ responsibility for protecting patient information and maintaining data security [Q19]. These accounts mainly reflected nurses’ roles as system users, information stewards, and contributors to incremental system improvement.

Higher-order clinical role cognition

Awareness of higher-order nursing informatics roles was present but unevenly developed. While most participants focused on operational responsibilities, a smaller subset articulated more advanced roles involving nurses’ contributions to information system improvement and development. For example, some students noted that nurses could provide opinions and suggestions for information system development based on their clinical experience [Q17] and contribute to developing systems better suited to clinical practice [Q18]. However, these higher-order perspectives were expressed less frequently and with less elaboration than operational roles. Participants rarely described specific involvement in system selection, evaluation, workflow optimization, knowledge dissemination, informatics leadership, or other strategic activities. Additionally, although students acknowledged the importance of continuous learning, few articulated concrete strategies for improving their own NI competence over time [Q20]. Overall, students’ awareness of higher-order nursing informatics roles appeared to be emerging but not yet systematically developed.

### 3.3. Synthesis of Quantitative and Qualitative Results

The integration of the quantitative and qualitative findings provided complementary evidence and greater contextual depth rather than simply demonstrating agreement between the two datasets (Table 10). The highly positive quantitative ratings of attitudes toward NI were broadly consistent with students’ qualitative accounts, which emphasized the value of digital technologies in improving efficiency, facilitating information access, and supporting patient care. At the same time, the qualitative findings extended these results by revealing students’ concerns that increasing reliance on digital technologies might compromise the humanistic aspects of nursing care. Thus, the qualitative data enriched, rather than merely confirmed, the survey findings.

Exploratory Spearman correlation analyses showed no significant positive associations between overall self-assessed NI competence and academic progression, previous exposure to information systems, or clinical experience. Although these findings should not be interpreted as causal evidence, they suggest that exposure alone may be insufficient to develop practical NI competence. Instead, the qualitative findings indicate that the educational quality, clinical relevance, and structure of learning experiences may be more influential, a proposition that warrants further investigation.

The qualitative findings also helped explain the relatively low quantitative scores for applied computer skills. Students consistently described limited opportunities for hands-on practice, predominantly passive learning approaches, and computer courses that were insufficiently integrated with nursing-specific tasks and clinical workflows. These experiences may help explain why students acquired basic technical knowledge but lacked confidence in applying informatics skills in clinical practice.

Similarly, while participants demonstrated a basic understanding of NI concepts and commonly identified operational roles, such as protecting patient information, managing clinical information, and providing feedback on information systems, a smaller subset also articulated higher-order responsibilities. These included contributing clinical opinions and suggestions to system improvement and participating conceptually in information system development [Q17, Q18]. However, such higher-order roles were expressed less frequently and with less conceptual elaboration than operational responsibilities, while workflow optimization, knowledge dissemination, and informatics leadership were seldom discussed. This pattern suggests that students’ awareness of advanced NI roles is emerging but remains uneven and not yet systematically developed.

Overall, the integrated findings indicate that students valued NI but reported gaps in applied competence, systematic conceptual understanding, and awareness of advanced professional roles. These findings further suggest that practical NI competence is unlikely to develop through academic progression or exposure to digital technologies alone. Instead, competence development may require structured educational scaffolding, authentic clinical learning opportunities, and stronger integration of informatics education with nursing practice. Because fourth-year students were not included in the qualitative strand, conclusions regarding their experiences during the transition to clinical practice should be interpreted cautiously.

## 4. Discussion

This mixed-methods study integrated self-assessed SANICS scores, exploratory correlation analyses, and qualitative accounts of experiences to examine the attitude–competence paradox among Chinese undergraduate nursing students. In the quantitative sample from two nursing schools, students reported highly positive attitudes toward NI but comparatively lower self-assessed applied competence. Qualitative accounts from Year 1–3 students at one participating school provided contextual insight into these patterns, particularly through descriptions of passive learning, general computer courses with limited clinical relevance, insufficient hands-on training, and few opportunities to apply informatics within nursing workflows. Because the quantitative and qualitative samples differed in their academic-year composition, the integrated findings should be interpreted as complementary contextual explanations rather than as evidence of complete convergence.

### 4.1. Interpreting the Attitude-Competence Paradox Through Situated Learning

The principal finding was a marked discrepancy between students’ positive attitudes toward NI and their lower self-assessed applied computer skills. The qualitative findings suggest that this discrepancy was not primarily attributable to a lack of interest in digital health. Students recognized the potential of digital technologies to improve information access, efficiency, and patient care. Their awareness of national digital health initiatives, including “Internet + Healthcare,” may also have reinforced these positive perceptions [9]. However, this awareness did not appear to be accompanied by corresponding levels of applied competence. This pattern suggests that familiarity with digital health policies may foster favorable attitudes, but it is insufficient on its own to prepare students for informatics-related clinical tasks.

Situated learning theory provides a useful lens through which to interpret this discrepancy. Lave and Wenger argue that professional competence develops through participation in socially and contextually meaningful communities of practice rather than through decontextualized knowledge acquisition alone [11]. Applied to NI education, this perspective suggests that students require opportunities to observe how informatics is used in nursing practice, rehearse relevant tasks, receive feedback, and gradually assume responsibility for informatics-related activities. Such activities may include electronic documentation, information-security decision-making, clinical decision support, and evaluation of digital workflows.

Participants’ accounts indicated that their learning often occurred through general computer instruction and passive knowledge acquisition, with limited connection to nursing-specific decisions and clinical workflows [23]. Rather than participating progressively in informatics-enabled nursing practice, students appeared to have few opportunities for legitimate peripheral participation within an authentic or realistically simulated nursing community of practice. In NI education, legitimate peripheral participation would involve students observing nurses utilizing EHRs, gradually progressing to entering data under supervision, and ultimately assuming responsibility for clinical documentation and decision-support tasks. The absence of such progressive participation in students’ accounts implies that NI learning remains largely decontextualized. This may help explain why students reported basic technical familiarity while expressing less confidence in applying informatics to clinical tasks.

Nevertheless, this interpretation should be approached with caution. This study did not include a formal curriculum audit, and participants were not systematically asked to describe the organizational structure of all informatics-related courses. The findings therefore support the presence of a perceived disconnect between general computer instruction and nursing practice, rather than establishing structural curriculum isolation as a definitive fact. The present study also did not systematically assess whether students had observational, simulated, supervised, or direct access to electronic health records or other nursing information systems during clinical placements. Although students perceived hands-on opportunities as limited, the precise nature, frequency, and extent of their participation in clinical information-system use remain unclear.

The exploratory correlation analyses provided additional context. Greater academic seniority and broader clinical exposure were negatively associated with attitudes toward NI, whereas neither variable was consistently associated with higher overall self-assessed competence. One possible explanation is that, as students progress academically and encounter digital systems in clinical settings, they become more aware of system limitations, usability problems, workflow disruption, or gaps between idealized digital-health expectations and actual clinical implementation. Less favorable attitudes may therefore reflect growing critical awareness rather than diminishing recognition of the importance of NI. Alternative explanations are also plausible, including cohort effects, variation in the quality of educational experiences, differences among clinical placement sites, and unmeasured confounding. Additionally, the negative association between clinical exposure and attitudes raises questions about the quality of students’ clinical informatics experiences. If students observe poorly designed or disruptive information systems during placements, they may develop a more critical perspective towards NI, even while their overall competence remains unaffected. This hypothesis warrants further qualitative investigation. Because clinical exposure was measured only as the number of exposure types reported, the present data cannot determine which specific experiences contributed to these associations. These findings should therefore be regarded as hypothesis-generating rather than as evidence of developmental or causal effects.

### 4.2. Positioning the Findings Within the International Literature

The present findings are broadly consistent with international studies in which nursing students reported moderate overall NI competence and particular weaknesses in applied skills [1,24]. Consistent with Guo et al.’s [10] survey of palliative care nurses in China, our results confirm that positive attitudes are not necessarily indicative of practical competence, suggesting that this pattern extends beyond the undergraduate population.

The educational contexts in which such discrepancies develop may nevertheless differ across institutions and countries. In the present study, participants described computer-related learning that was insufficiently connected to nursing practice. International competency frameworks, including the Technology Informatics Guiding Education Reform (TIGER) initiative [25], increasingly recommend longitudinal integration of informatics competencies across nursing curricula rather than reliance on isolated technical instruction. However, the extent to which these recommendations have been implemented varies across educational programs. Therefore, the current findings should not be interpreted as demonstrating a simple distinction between Chinese and Western curricula. Instead, they highlight the need to examine how the organization, clinical relevance, and practical intensity of informatics education vary across settings. In the Chinese context, large student cohorts and restricted access to hospital information systems during clinical placements may exacerbate the theory-practice gap observed in this study. These structural constraints warrant targeted educational innovations, such as virtual Electronic Health Record (EHR) platforms and simulation-based learning, as discussed below.

Students’ awareness of Chinese national digital health policies represents a potentially important contextual feature. Such awareness may help explain their highly positive attitudes toward NI. However, the coexistence of positive attitudes and lower applied competence indicates that policy familiarity alone may not produce practice readiness. Translating national digital health priorities into undergraduate competence is likely to require implementation-focused education that provides repeated, supervised opportunities to apply informatics within nursing-specific contexts.

### 4.3. Translating Evidence into Action: Curriculum Reform and Simulation Strategies

To bridge this gap, nursing education must address practical barriers, such as large class sizes and limited access to clinical information systems. We propose an integrated approach: first, curriculum integration must serve as the structural foundation. Nursing programs should thread NI concepts throughout existing courses (e.g., incorporating data security into ethics, medication alerts into pharmacology) [23,25]. Second, programs should utilize virtual EHR platforms and low-cost digital documentation exercises within clinical skills labs to provide authentic practice environments [26]. Finally, project-based learning—such as mapping clinical workflows—can help students apply these concepts and develop higher-order informatics roles beyond basic tool operation [27].

### 4.4. Educational and Policy Implications for Chinese Nursing Programs

The findings support an integrated pathway in which curricular content, simulation, assessment, and clinical participation function as complementary components of NI competence development. Rather than treating curricular integration, simulation, and project-based learning as separate strategies, nursing programs could organize them as successive and mutually reinforcing stages of learning.

First, NI content could be threaded longitudinally through existing nursing courses rather than being confined to stand-alone computer instruction. Information privacy and cybersecurity could be incorporated into nursing ethics; medication alerts and clinical decision support into pharmacology; and electronic documentation, data quality, and digital handover into fundamental and medical–surgical nursing courses [25,28]. A progressive curriculum could move from foundational digital literacy to electronic documentation, clinical decision support, data ethics, system evaluation, and workflow improvement across the undergraduate program.

Second, integrated content should be reinforced through repeated opportunities for simulation and performance-based assessment. Virtual EHR platforms, simulated patient records, and low-cost digital documentation activities could be incorporated into clinical skills laboratories, particularly where access to hospital information systems is restricted. Students could be assessed through clinically meaningful tasks, such as documenting a simulated patient encounter, responding to a medication alert, identifying unsafe information-sharing practices, or evaluating the usability of a digital nursing workflow. Project-based assignments, including workflow mapping or redesign of a documentation process, could further support students’ transition from basic tool use to higher-order informatics roles involving system evaluation and quality improvement [29].

Third, academic–clinical partnerships are needed to facilitate transfer from classroom and simulated learning to clinical practice. Clinical partners could provide supervised observation, sandbox environments, or training-mode access to selected information-system functions while protecting patient privacy and safety. Such arrangements may be particularly relevant in Chinese educational settings characterized by large student cohorts, limited clinical placement capacity, and variable access to hospital information systems. Shared simulation platforms, standardized digital clinical exercises, and interinstitutional resource sharing may help reduce these implementation barriers.

These implications involve multiple stakeholders. Educators should align teaching and assessment with clinically meaningful tasks rather than relying predominantly on written examinations. Curriculum developers should establish a progressive NI learning pathway and map informatics outcomes across core nursing courses. Clinical partners should clarify the level of supervised information-system participation that is appropriate for students during placements. Policymakers and professional organizations could consider developing nationally aligned undergraduate NI competency expectations, accompanied by guidance on curriculum mapping and performance-based assessment. Although students may benefit from workshops, seminars, and practice-oriented digital health projects, individual initiative should complement rather than substitute for structured curricular and clinical learning opportunities.

### 4.5. Study Limitations

Several limitations should be acknowledged. First, this study was conducted in two public undergraduate nursing schools in metropolitan Guangzhou, and the findings may not transfer directly to rural programs, private institutions, or regions with different educational and technological resources. Second, the cross-sectional design precludes causal inference. Third, SANICS measured self-assessed rather than objectively demonstrated competence; students may have overestimated or underestimated their abilities, and the observed attitude–competence discrepancy may differ from a gap measured using performance-based assessments. Objective, factual, and conceptual knowledge was not measured quantitatively. Therefore, mixed-methods integration within the knowledge dimension relied primarily on qualitative accounts of students’ conceptual understanding. Focus groups may also have been affected by social desirability and group-interaction effects, and the qualitative findings reflect students’ perceptions rather than direct observation of their clinical performance or curriculum delivery. The qualitative strand included students from one school and did not include fourth-year students because they were undertaking clinical placements. This limited insight into the transition-to-practice stage and reduced comparability between the quantitative and qualitative samples. In addition, this study did not capture the frequency, duration, quality, supervision, or level of active participation associated with students’ use of clinical information systems. The exploratory Spearman analyses involved selected educational variables and should be interpreted as contextual associations rather than causal predictors. Finally, the sample size was determined using an item-to-participant heuristic rather than a formal power analysis. Future hypothesis-testing and predictive studies should use a priori power calculations.

### 4.6. Future Directions

To build upon the current findings, future research must transition from cross-sectional, self-report designs to adequately powered, hypothesis-driven methodologies. Longitudinal studies are particularly needed to track how students’ NI competence and attitudes evolve as they progress from preclinical coursework into clinical placements. Future qualitative and mixed-methods research should also align sampling more closely to ensure the inclusion of fourth-year students, thereby capturing the critical transition to professional practice. Furthermore, future studies must evaluate NI education using objective measures to overcome the inherent biases of self-assessment. Researchers should incorporate validated objective knowledge assessments-or develop and psychometrically evaluate such instruments where appropriate measures are currently unavailable. It is crucial to compare self-assessed competency scores with objective, performance-based tasks, such as simulated EHR documentation, medication-barcode verification, data-security decision-making, and the use of clinical decision-support systems. In addition, measurement tools must capture clinical exposure with greater granularity. Rather than recording exposure merely as present or absent, future instruments should distinguish the frequency, duration, quality, and level of active participation associated with students’ use of nursing information systems. Finally, multi-center interventional studies are warranted to rigorously test the effectiveness of targeted educational strategies. Evaluating the impact of embedded digital documentation modules, EHR simulation platforms, and project-based system-improvement assignments across diverse nursing programs will provide critical evidence for establishing best practices in undergraduate NI education.

## 5. Conclusions

This mixed-methods study contributes to the NI literature by moving beyond the description of competence levels to provide contextual insights into the attitude–competence paradox among Chinese undergraduate nursing students. By integrating self-assessed competence and attitude data with students’ accounts of their learning experiences, this study identified a pattern in which highly positive attitudes toward NI coexisted with comparatively lower applied competence. The qualitative findings suggest that passive learning approaches, limited hands-on practice, and a perceived disconnect between general computer instruction and nursing-specific clinical workflows may help explain this discrepancy. These findings provide context-specific evidence to inform more targeted curriculum development in Chinese undergraduate nursing education. The findings further indicate that positive attitudes, academic progression, and exposure to digital technologies alone may be insufficient to ensure readiness for informatics-enabled practice. Therefore, NI education should be integrated longitudinally across core nursing courses and reinforced through simulation, performance-based assessment, and supervised participation in authentic clinical contexts. As healthcare becomes increasingly digital, developing NI competence is not merely an educational enhancement but an essential requirement for preparing future nurses to deliver safe, efficient, and high-quality care.

## Figures and Tables

**Table 1:** Operational definitions and measurement of the core study concepts.

Concept	Operational Definition in This Study	Measurement Source	Measurement Approach
Knowledge	Students’ conceptual understanding of nursing informatics and digital health, including definitions, principles, common applications, and related technologies. This refers to expressed understanding rather than objectively tested factual knowledge.	Focus group interviews	Inductive thematic analysis of students’ descriptions and explanations
Perceptions	Students’ beliefs, attitudes, evaluations, and judgments regarding nursing informatics and digital health, including perceived value, usefulness, limitations, and future development.	SANICS attitudes subscale + focus groups	Self-assessment questionnaire and thematic analysis
Skills	Students’ self-perceived ability to use nursing informatics technologies and digital tools in learning and clinical settings. Skills refer to perceived competence rather than objectively demonstrated performance.	SANICS competence domains + focus groups	Self-assessment questionnaire supplemented by qualitative reports of learning and application experiences

Note. SANICS: Self-assessment of Nursing Informatics Competencies Scale.

**Table 2:** Domains in the Self-assessment of Nursing Informatics Competencies Scale.

Domain	Explanation	Examples of Scale Items
Role of clinical informatics	The practice that nurses implemented in the clinical informatics	Promote the integrity of and access to information to include but not limited to confidentiality, legal, ethical, and security issues
Basic computer knowledge and skills	Techniques and tools in systems analysis and project management	Use database management program to develop a simple database and/or table
Applied computer skills	Use of computer hardware and software	Perform basic troubleshooting in applications
Wireless device skills	Wireless device to locate resources and enter data	Use wireless device (PDA or cellular telephone) to enter data
Nursing informatics attitudes	Evaluations on NI	Recognize that Internet + health will become more commonplace

**Table 3:** Demographic characteristics of participants (*N* = 149).

Item	Subgroup	Number of Students	Percentage (%)
Age	<21 years old	41	27.1
	21–24 years old	105	70.4
	≥24 years	3	2
Sex	Male	16	10.7
	Female	133	89.3
Year	Year 1	9	6
	Year 2	15	10.1
	Year 3	26	17.4
	Year 4	99	66.4
Equipment touched (Multiple choice)	Computer	141	24.4
	Smartphone	144	24.9
	Tablet	118	20.4
	Medical Information System	67	11.6
	Online Learning System	105	18.1
	Other	4	0.7
Daily revision time after class	<30 min	42	28.2
	30 min–1 h	63	42.3
	≥1 and <2 h	24	16.1
	≥2 and <3 h	13	8.7
	≥3 h	7	4.7
Computer Levels Earned	None	80	53.7
	Level 1	34	20.1
	Level 2	52	30.8
	Level 3	2	1.2
	Level 4	1	0.6
Channels for learning professional knowledge	Books	133	34.2
	Website	134	34.4
	Online Systems	118	30.3
	Other	4	1
Have you studied or been exposed to nursing information systems	Yes	119	79.9
	No	30	20.1

Note. For single-response variables, percentages were calculated using *N* = 149 as the denominator. For multiple-response variables, percentages were calculated using the number of responses for that item as the denominator; therefore, totals may exceed 100% when interpreted at the respondent level.

**Table 4:** Nursing informatics competence scores (*N* = 149).

Dimension	Total Score (Mean ± SD)	Item Mean (Mean ± SD)	Cronbach’s Alpha
Clinical informatics role	11.94 ± 3.68	2.39 ± 0.74	0.912
Basic computer knowledge and skills	31.01 ± 7.20	2.82 ± 0.65	0.943
Applied computer skills	8.07 ± 3.21	2.02 ± 0.80	0.919
Wireless device skills	10.07 ± 3.09	2.52 ± 0.77	0.906
Attitudes toward NI	16.66 ± 3.06	4.16 ± 0.76	0.906
Total scale	77.74 ± 15.2	2.78 ± 0.54	0.946

Note. Cronbach’ alpha was calculated using the original item-level data from the present sample.

**Table 5:** Top five and bottom five individual items (*N* = 149).

Item	Mean ± SD	Dimension	Rank
Awareness of the importance of clinician involvement in the design, selection, implementation and evaluation of healthcare systems	4.21 ± 0.83	NI attitudes	1
Recognize that computer programmers are not the only ones who can effectively apply computer technology to nursing care	4.19 ± 0.83	NI attitudes	2
Recognize that “internet + health” will become more commonplace	4.17 ± 0.82	NI attitudes	3
Recognize that computers are only a tool to facilitate nursing care and cannot completely replace human functions.	4.09 ± 0.97	NI attitudes	4
Creating slides (e.g., PPT) using word processing, presentation graphics, and presenting with multimedia	3 ± 0.75	Basic computer knowledge and skills	5
Identify basic components of a computer system (e.g., functions of a PC, workstation).	2.21 ± 0.86	Applied computer skills	24
Being a leader and being able to integrate innovative concepts and informatics concepts into one’s area of expertise	2.2 ± 0.87	Clinical informatics role	25
Perform basic troubleshooting in applications	2.13 ± 0.91	Applied computer skills	26
Use apps for diagnostic coding	1.87 ± 0.91	Applied computer skills	27
Develop test materials using applications	1.85 ± 0.91	Applied computer skills	28

**Table 6:** Exploratory Spearman correlations between selected educational variables and SANICS scores (*N* = 149).

Variable	Total	Clinical Informatics Role	Basic Computer Knowledge and Skills	Applied Computer Skills	Wireless Device Kills	Attitudes Toward NI
Year of study	ρ = −0.113, *p* = 0.170	ρ = −0.061, *p* = 0.456	ρ = −0.132, *p* = 0.108	ρ = 0.047, *p* = 0.568	ρ = 0.010, *p* = 0.900	ρ = **−0.331**, ***p* < 0.001**
Prior exposure to nursing information systems	ρ = −0.077, *p* = 0.351	ρ = −0.107, *p* = 0.195	ρ = −0.112, *p* = 0.174	ρ = −0.001, *p* = 0.989	ρ = 0.058, *p* = 0.480	ρ = −0.107, *p* = 0.194
Computer course experience	ρ = −0.115, *p* = 0.164	ρ = **−0.199**, ***p* = 0.015**	ρ = −0.100, *p* = 0.224	ρ = −0.146, *p* = 0.075	ρ = −0.031, *p* = 0.705	ρ = **0.217**, ***p* = 0.008**
Clinical exposure level	ρ = −0.150, *p* = 0.069	ρ = −0.006, *p* = 0.938	ρ = −0.110, *p* = 0.182	ρ = 0.055, *p* = 0.503	ρ = 0.023, *p* = 0.783	ρ = **−0.279**, ***p* < 0.001**

Note. Values are Spearman’s rho and two-tailed *p* values. Prior exposure to nursing information systems and computer-course experience were coded as 0 = no and 1 = yes. Clinical exposure was represented by the original clinical exposure score in the dataset, with higher values indicating more types of clinical exposure reported. Significant correlations are shown in bold.

**Table 7:** General demographic information (*n* = 23).

ID	Age	Sex	Grade	Place of Birth	Computer Courses	Avenues for Digital Health Knowledge	Use of Nursing Information System	Clinical Practice
1a	19	Male	Freshman	Urban	Yes	Books, websites, online systems	Yes	Yes
1b	20	Female	Sophomore	Rural	Yes	Books, websites, online systems, communication with teachers	Yes	Yes
1c	21	Female	Sophomore	Rural	Yes	Books, websites, online systems	No	Yes
1d	21	Female	Sophomore	Town	Yes	Books, websites, online systems	No	Yes
2a	19	Female	Freshman	Rural	Yes	Books, websites	No	Yes
2b	20	Female	Sophomore	Rural	Yes	Books, websites, online systems	No	Yes
2c	20	Female	Third year	Rural	Yes	Books, websites, online systems, APP	Yes	Yes
2d	19	Female	Freshman	Town	Yes	Books, websites, classes	Yes	Yes
3a	19	Female	Freshman	Town	Yes	Books, websites, online systems, professional papers	No	Yes
3b	20	Male	Sophomore	Town	Yes	Books, websites, online systems	No	Yes
3c	20	Female	Freshman	Urban	Yes	Books, websites, online systems	Yes	Yes
4a	20	Female	Sophomore	Rural	Yes	Books, websites, online systems	Yes	Yes
4b	20	Female	Sophomore	City	Yes	Books, websites, online systems	No	Yes
4c	19	Female	Freshman	Rural	Yes	Books, websites	No	Yes
4d	20	Female	Freshman	Urban	Yes	Books, websites, classes	No	Yes
5a	19	Female	Freshman	Urban	Yes	Books, websites, online systems	No	Yes
5b	19	Male	Sophomore	Town	Yes	Books, websites, online systems	Yes	Yes
5c	20	Female	Third year	Rural	Yes	Books, websites, online systems	No	Yes
5d	20	Female	Sophomore	Town	Yes	Books, websites, online systems	Yes	Yes
6a	19	Female	Freshman	Rural	Yes	Books, websites, online systems, classes	Yes	Yes
6b	20	Female	Sophomore	Rural	Yes	Books, websites, online systems, classroom lectures, seminars	Yes	Yes
6c	21	Female	Sophomore	Town	Yes	Books, websites	Yes	Yes
6d	20	Female	Sophomore	Rural	Yes	Books, websites, online systems, Clove Garden	No	No

**Table 8:** Simplified coding framework of qualitative themes.

Themes	Content	Subthemes
Theme 1: Knowledge of NI and digital health	Cognitive understanding of NI and digital health, including definitions, disciplinary attributes, policies, application principles, and clinical tools	▪ Conceptual understanding ▪ Application tools
Theme 2: Perceptions of NI and digital health	Subjective attitudes and cognitive judgments regarding the value, development trends, and limitations of NI and digital health	▪ Recognition of application value ▪ Awareness of limitations
Theme 3: Skills and clinical role cognition	Practical abilities to apply NI and digital health technologies in nursing practice, covering basic tool mastery, higher-order application, and innovation capabilities	▪ Skill acquisition pathways ▪ Basic clinical role cognition ▪ Higher-order clinical role cognition

**Table 9:** Representative quotations by theme and subthemes.

Theme	Subtheme	Representative Quotation
Theme 1: Knowledge of NI and digital health	Conceptual understanding	Q1: “The core competencies of nursing informatics lie in the use of computers and the analysis and retrieval of information.” (Student 5c)
		Q2: “Digital nursing is an interdisciplinary field integrating computer science and nursing science.” (Student 3c)
		Q3: “We gained a superficial understanding of digital nursing through several ‘Internet +’ research projects.” (Student 5c)
		Q4: “Nursing informatics offers remote care and health counseling for patients at home via Internet platforms.” (Student 3a)
		Q5: “The computerization of nursing care requires the protection of patients’ privacy.” (Student 2a)
		Q6: “The computerization of care must follow the principle of respect; patients must participate voluntarily.” (Student 5b)
		Q7: “The accuracy of the information is crucial, and the authenticity of patient information must be verified.” (Student 2b)
	Application tools	Q8: “The widespread use of electronic patient records underscores the importance of electronic information technology in nursing work.” (Student 1a)
		Q9: “PDA devices can scan codes to verify patient identities and corresponding medications.” (Student 3b)
		Q10: “Healthcare informatics enables remote care for diabetic patients living at home via internet platforms.” (Student 3a)
Theme 2: Perceptions of NI and digital health	Recognition of application value	Q11: “The biggest advantage of digital health is that it saves time and effort, reducing the workload of medical staff.” (Student 2d)
		Q12: “The computerization of nursing benefits both doctors and patients, improving work efficiency and giving nurses more time to care for patients.” (Student 3b)
	Awareness of limitations	Q13: “Nursing care must also offer patients psychological and humanistic care, which needs to be further strengthened.”(Student 4c)
Theme 3: Skills and clinical role cognition	Skill acquisition pathways	Q14: “Through courses and lectures, I have gained some understanding, but only at a superficial level.” (Student 5c)
		Q15: “The C# learned in computer science class is impractical; the Python learned by second-year students is more accessible, but I only dabbled in it.” (Student 2b)
	Basic clinical role cognition	Q16: “We are beneficiaries of nursing computerization and providers of feedback to point out irrational design and drive program optimization.” (Student 2c)
		Q17: “We can contribute opinions and suggestions to the development of information systems based on our clinical experience.” (Student 5b)
	Higher-order clinical role cognition	Q18: “Nurses have professional knowledge and, compared to programmers, can contribute to developing a system better suited to clinical practice.” (Student 5c)
		Q19: “Nurses are the stewards of nursing information and need to ensure the security of patient information.” (Student 1a)
		Q20: “As learners, nurses must have knowledge of nursing informatics, basic computer skills, and information management skills.” (Student 3a)

**Table 10:** Integration of quantitative and qualitative findings across core dimensions.

Dimension	Quantitative Finding	Qualitative Finding	Interpretation
Knowledge	Objective knowledge was not directly measured; SANICS provided self-assessed competence and attitude data.	Students described NI using basic functional definitions [Q1–Q2] and named common digital tools [Q8–Q10], but did not elaborate on interdisciplinary or higher-order concepts.	Qualitative data provided the main evidence for students’ conceptual understanding; students demonstrated basic familiarity with NI definitions and common tools, but their qualitative accounts revealed a limited understanding of interdisciplinary concepts, system optimization, data-driven decision-making, or higher-order clinical applications. Quantitative data should not be interpreted as an objective knowledge test.
Perceptions	Attitudes toward NI scored highest (4.16 ± 0.76).	Students recognized the value of NI in improving efficiency [Q11–Q12]; a few noted concerns about humanistic care [Q13].	Qualitative findings confirmed the highly positive attitudes captured in the survey, while adding critical nuance by revealing students’ underlying concerns about maintaining humanistic care in increasingly digital environments.
Skills	Overall self-assessed competence was slightly below the midpoint of the scale (2.78 ± 0.54); applied computer skills scored lowest (2.02 ± 0.80).	Students reported passive learning pathways and theory-practice disconnect [Q14–Q15]. Basic roles (feedback, security) were recognized [Q16, Q19]; higher-order roles were less frequently articulated [Q17–Q18, Q20].	Qualitative findings not only confirmed the low applied skill scores but also revealed why these skills remain underdeveloped, specifically highlighting passive learning experiences, impractical curricula, and limited hands-on clinical training. These are plausible explanations rather than statistically tested predictors.

## Data Availability

The data presented in this study are available upon reasonable request from the corresponding author, subject to ethical restrictions.

## Supplementary Materials

The following supporting information can be downloaded at: https://www.mdpi.com/article/10.3390/nursrep16080268/s1, Additional File S1: Interview Guide; Additional File S2: Expanded qualitative quotation tables.

## Public Involvement Statement

No public involvement in any aspect of this research.

## Guidelines and Standards Statement

This manuscript was drafted in accordance with STROBE (Strengthening the Reporting of Observational Studies in Epidemiology) guidance for cross-sectional research and COREQ (Consolidated Criteria for Reporting Qualitative Research) guidance for qualitative reporting.

## Abbreviations

The following abbreviations are used in this manuscript:

NI	nursing informatics
AACN	American Association of Colleges of Nursing
SANICS	Self-assessment of Nursing Informatics Competencies Scale
PDA	personal digital assistant
PBL	project-based learning
EHR	electronic health records
TIGER	Technology Informatics Guiding Education Reform
