# Supplementary material for: Nursing Informatics Competence and Digital Health Readiness Among Chinese Undergraduate Nursing Students: A Convergent Mixed-Methods Study
Source: Nurs Rep. 2026 Jul 31;16(8):268. doi: 10.3390/nursrep16080268 (PMC13517043; doi:10.3390/nursrep16080268)
Supplement: Supplementary file 1 [file nursrep-16-00268-s001.zip › nursrep-4388518-supplementary.pdf]

Additional file S1: Interview Guide

Interview Guide
1. Introductions (1 minute)
Self-introductions from the researcher and interviewee.
2. Please describe your understanding of nursing informatics or digital health.
(Research focus: definitions, development, core content (public policy, data management, patient safety), ethical principles (data security, privacy), and related technologies (smart hospital systems, e-health innovations))
3. What channels have you used to learn about nursing informatics or digital health?
(Research focus: sources of knowledge, e.g., coursework, clinical practice, self-study)
4. How do you perceive the importance of nursing informatics or digital health for medical and nursing practice? Please provide examples.
(Research focus: impacts of public policy on care delivery, and the role of nursing informatics/digital health in clinical settings, e.g., smart hospital nursing systems)

Additional file S2: Expanded qualitative quotation tables

**Theme**	**Subtheme**	Representative Quotation
Theme 1: Knowledge of Nursing Informatics(NI) and Digital Health	Conceptual understanding and core concepts of nursing informatics	Q1. *Digitizing nursing information reduces labor costs and the error rate when sorting patient data, standardizes the storage of patient information, and improves the quality of nursing services from the patient's perspective.(Student ID：3d)*<br>Q2. *The core competencies of nursing informatics lie in the use of computers and the analysis and retrieval of information. These skills must be acquired through the study of nursing information in order to apply them in digital healthcare and provide better care for patients.(Student ID：5c)*<br>Q3. *Essentially, this means comparing the relevant data obtained with normal indicators in order to assess the actual effect of medical interventions or related measures.(Student ID：7b)*<br>Q4. *Digital nursing is an interdisciplinary field of digital computer science and nursing science that integrates the advantages of both areas to compensate for their respective weaknesses. Its core consists of strengthening nursing science with digital information, closing gaps in its development, and promoting the advancement of the discipline.(Student ID：3d)*<br>Q5. *Patient data is processed using technical means in order to optimize the quality of care services.(Student ID：4a)*<br>Q6. *Digital nursing tends toward database management of patient data, with a focus on the uniform management and planning of this data.(Student ID：6a)*
	Policy context and healthcare development	Q7. *We began scientific research in our second and third years of study and gained a superficial understanding of digital nursing through several "Internet +" research projects supervised by teachers.(Student ID:5c)*<br>Q8. *The 19th National Congress proposed the*

Theme	Subtheme	Representative Quotation
		"Internet + Health" development concept to promote national health and support the provision of health services to the public through appropriate systems.(Student ID:6a)<br>Q9. *Under the influence of national strategies such as "Smart Healthcare" and "Healthy China," the healthcare sector is evolving toward "Internet +."(Student ID:7c)*
	Clinical applications and healthcare services	Q10. *I will be taking the elective course "Intelligent Mobile Care" next semester. This field relies on nursing informatics to offer remote care, health counseling, and family care interventions for patients at home via Internet platforms, with the "Sugar Mom Platform" being a typical application. Home care can also be implemented with this technology.(Student ID:3b)*<br>Q11. *During my probationary period at the Third Affiliated Hospital, I observed my mentor entering all the information about a newly admitted patient,including the care plan, into the computer to create a complete patient record that was updated and tracked daily.(Student ID:3d)*
	Ethical principles and information governance	Q12. *The computerization of nursing care requires the protection of patients' privacy and the non-disclosure of their personal data in the system at will, which is what our teachers also taught us during our trial period at the hospital.(Student ID:2e)*<br>Q13. *An important point when combining care and computerization is the confidentiality of information. When using basic patient data, the principle of confidentiality must be observed in order to prevent information leaks.(Student ID:4b)*<br>Q14. *Protecting patient privacy is an important principle. Various apps currently have the problem of stealing personal data, and patients' health data must not be misused.(Student ID:5b)*

Theme	Subtheme	Representative Quotation
		Q15. *Network problems can lead to the theft and sale of patients' personal data, and there is also a potential risk of medical technology being stolen.(Student ID:7b)*<br>Q16. *The computerization of care must also follow the principle of respect; patients must participate voluntarily in the relevant processes and must not be forced to do so.(Student ID:5b)*<br>Q17. *When implementing computerization measures in the care sector, patients' wishes should be respected. Even if a rehabilitation or treatment plan is drawn up based on their information, patients cannot be forced to implement it if they reject it for conceptual reasons.(Student ID:6b)*<br>Q18. *Patients' personal wishes should be respected when entering their data, and non-essential information can be omitted as necessary. When it comes to important information, nursing staff must fulfill their communication obligations properly.(Student ID:7e)*<br>Q19. *In light of the computerization of nursing care, recorded patient information should be concise and accurate.(Student ID:2d)*<br>Q20. *The accuracy of the information is crucial, and the authenticity of the uploaded patient-related information must be verified.(Student ID:2b)*<br>Q21. *The most important prerequisite for data collection is ensuring authenticity and accuracy. Just as with the problem of false information in questionnaire surveys, the subsequent related work cannot be carried out if the data is falsified.(Student ID:3d)*
	Technological tools and digital applications	Q22. *.In today's information age, the widespread use of electronic patient records and electronic care records underscores the importance of electronic information technology in nursing work.(Student ID:1a)*<br>Q23. *Our teacher introduced a hospital tool*

Theme	Subtheme	Representative Quotation
		that allows doctors to log in to their accounts and check on the condition of inpatients in real time from home, enabling them to monitor their patients' progress even during their free time, which is very convenient.(Student ID:7d)<br>Q24. *Through my research, I have found that nursing informatics is more mature in management applications in areas such as intensive care and surgery, which is reflected in the use of electronic patient records.(Student ID:3d)*<br>Q25. *The wristbands and PDA devices worn by inpatients can scan codes to verify patient identities and corresponding medications, replacing the previous method of inquiry and verification.(Student ID:3c)*<br>Q26. *When family members require medical treatment, they can make doctor's appointments online via mini-programs from hospitals, official accounts, or apps.(Student ID:7d)*<br>Q27. *Healthcare informatics enables remote care for diabetic patients living at home. For example, the Sugar Mom Platform at Third Hospital can offer patients health advice and family care interventions via the internet, and the daily care of ordinary diabetic patients can also be provided in this way.After surgery, patients can scan QR codes to watch short instructional videos on rehabilitation and receive relevant rehabilitation instructions.(Student ID:3b)*<br>Q28. *When I was volunteering at the hospital, I realized that the smart little machines in the hospital could help medical visitors plan their routes.(Student ID:6a)*<br>Q29. *Sports wristbands can monitor data such as heart rate and blood pressure in real time and synchronize it with mobile phone apps that not only analyze the data but, in some cases, also offer exclusive health care features.(Student ID:5c)*

**Theme**	**Subtheme**	Representative Quotation
Theme 2: Perceptions of NI and digital health	Learning pathways and knowledge acquisition	Q30. *I have previously studied medical statistics, but I have not yet mastered it sufficiently.(Student ID:2d)*<br>Q31. *Through medical statistics, courses in nursing research, and Nanshan lectures, I have gained some understanding of relevant content, but only at a superficial level.(Student ID:5c)*<br>Q32. *I gained insights from internet projects for seniors, the application of the internet in various national areas, and changes in life, and then thought about how this could be combined with the care sector.(Student ID:5b)*<br>Q33. *I acquired relevant knowledge by reading specialist articles, participating in innovation projects, and studying the connection between the Internet and nursing in the course "Introduction to the Thoughts of Mao Zedong." (Student ID:Fc)*<br>Q34. *The C# I learned in computer science class is impractical and confuses me; the Python learned by second-year students is more accessible, but I only dabbled in it a little and didn't understand it.(Student ID:Cb)*<br>Q35. *During the probationary period, teachers provide opportunities to collect medical records in order to practice communication between doctor and patient, and take us to review patients' medical records after collection, giving me a general understanding of the process.(Student ID:1a)*<br>Q36. *During my probationary period, I became familiar with various medical record systems.(Student ID:2c)*<br>Q37. *During my probationary period, I saw teachers entering a lot of information into the computer after visits or operations, and after the teachers showed us around, I found this method very practical.(Student ID:4d)*<br>Q38. *The 19th National Congress proposed the "Internet + Health" development concept, which aims to promote national health and support the provision of health services to the*

**Theme**	**Subtheme**	Representative Quotation
		public through various systems.(Student ID:6c)
		Q39. *The biggest advantage of digital health is that it saves time and effort, which can reduce the workload of medical staff.(Student ID:2e)*
		Q40. *The computerization of nursing care benefits both doctors and patients. It can reduce unnecessary work and paperwork, improve work efficiency, and give nursing staff more time to care for patients.(Student ID:3c)*
		Q41. *The computerization of nursing applies data-based technologies to nursing processes. Currently, hospitals still use some paper-based methods for data entry, which consume staff and energy. Future computerization will facilitate the collection of patient information, case management, and the implementation of nursing processes.(Student ID:4b)*
	Recognition of clinical and professional value	Q42. *Healthcare informatics and digital health have promoted the development and advancement of the medical industry to a certain extent.(Student ID:7b)*
		Q43. *The computerization of nursing enables uniform classification and management of patient information, facilitates nursing diagnosis and scientific analysis and sorting of medical information, which not only benefits patient health, but can also improve the efficiency of hospital operations and doctors' diagnoses.(Student ID:6d)*
		Q44. *After applying nursing information technology in clinical practice, patient feedback can be forwarded to supervisors.(Student ID:7d)*
		Q45. *Nursing staff must be able to collect, obtain, and process information in order to carry out nursing diagnoses and other related nursing tasks using this information from a nursing perspective.(Student ID:5d)*
		Q46. *Digital health can improve the accuracy of data entry. Similar to the input help for missing questionnaire information, there will*

**Theme**	**Subtheme**	Representative Quotation
		also be such accuracy guarantees for the entry of patient records.(Student ID:2e)<br>Q47. *The integration of Internet technology can improve the accuracy and processing efficiency of extensive basic patient information.(Student ID:4c)*<br>Q48. *The scientific analysis and sorting of patient information in medicine is not only of great benefit to patients' health, but can also significantly improve the efficiency of internal hospital processes and diagnosis by physicians.(Student ID:6d)*<br>Q49. *The computerization of healthcare enables patients to develop a clearer understanding of diseases and medical treatment options and to acquire systematic knowledge about the entire process of diagnosis and treatment of diseases as well as postoperative rehabilitation, which is far better than before.(Student ID:3c)*
	Awareness of limitations and humanistic care needs	Q50. *Nursing care must not only ensure the physical care of patients with professional techniques, but also offer them psychological, humanistic care, which needs to be further strengthened in the field of humanistic nursing.(Student ID:4c)*
Theme 3: Skills and clinical role cognition	Feedback provision and system improvement	Q51. *We are both beneficiaries of the computerization of nursing, whose design streamlines and facilitates our work, and providers of feedback who can point out irrational design aspects in order to drive program optimization forward.(Student ID:2c)*<br>Q52. *While enjoying the benefits of informatization in healthcare, we must identify and resolve problems in a timely manner to contribute to its promotion and better development. The development of this system is closely linked to us.(Student ID:3d)*<br>Q53. *We can be both users and feedback providers for the computerization of nursing care and give feedback on its shortcomings in order to drive its improvement and*

**Theme**	**Subtheme**	Representative Quotation
		perfection.(Student ID:4e)<br>Q54. *After identifying the problems and areas for improvement in the computerization of nursing care, we consider adaptation plans and pass these on to the technicians so that they can perfect the relevant technologies and equipment.(Student ID:5d)*<br>Q55. *We evaluate the computerization of nursing care before and after its introduction and draw conclusions. Problems are continuously identified and improved during the process, and comparisons in subsequent nursing care show that improving measures not only saves time but also leads to qualitative improvements.(Student ID:6b)*
	Participation in system selection, design, implementation, and evaluation	Q56. *We can contribute relevant opinions and suggestions to the development and improvement of information systems based on our expertise and clinical experience.(Student ID:7b)*<br>Q57. *Technicians are more professional in programming and improving medical devices. Once we have acquired the relevant technical knowledge, we can make optimization suggestions to make the relevant technologies and devices more user-friendly for nursing work, which we then apply in patient care.(Student ID:5d)*<br>Q58. *Nursing staff must have the appropriate skills for gathering, recording, and processing information, and the importance of nursing work is also reflected in the linking of information processing.(Student ID:5c)*<br>Q59. *Evaluate and draw conclusions before and after implementing nursing measures, continuously identify and improve problems in the process, and comparisons in subsequent care show that optimizing measures can not only save time but also achieve qualitative improvements.(Student ID:6d)*
	Integration of	Q60. *Nurses can enter patients' basic*

**Theme**	**Subtheme**	Representative Quotation
	informatics into nursing practice	*information while providing care, retrieve medical histories, and forward them to doctors. They can also use the information system to communicate with senior nurses, head nurses, or doctors if they encounter difficult situations during the night shift handover.(Student ID:7e)* Q61. *Nurses can take on a wide range of tasks in the treatment of chronic diseases, geriatric care, and other areas. In addition, their work can lay the foundation for the formulation of medical guidelines and even influence political decisions.(Student ID:7d)*
	Professional roles and responsibilities in nursing informatics	Q62. *In addition to performing nursing tasks, nurses must also provide health education. They can familiarize patients with the relevant system and show them how to use it correctly to support their recovery.(Student ID:2b)* Q63. *Nurses have professional medical and nursing knowledge and, compared to pure programmers, can contribute to the development of a nursing information system that is better suited to clinical practice and more convenient and efficient.(Student ID:5c)* Q64. *I once learned that President Zhou's research project was a collaboration between nurses and the social insurance sector, in which nurses were involved in research on insurance enrollment and the choice of insurance rates for patients.(Student ID:7c)* Q65. *Nurses are the stewards of nursing information, who not only need to enter the details of patient information, but also need to ensure the security of patient information and prevent information leaks.(Student ID:1a)* Q66. *Compared to the previous paper-based administration system with extremely low error tolerance, digital administration has higher error tolerance and a lower error rate, which significantly improves patient medication safety, care safety, and other aspects.(Student ID:1a)* Q67. *The computerization platform can clearly*

**Theme**	**Subtheme**	Representative Quotation
		illustrate the implementation effects of care measures and compare the effects before and after each improvement in order to assess the effectiveness, rationality, and practicability of the improvement. This facilitates feedback and reflection and makes the recording and improvement of the care process traceable.(Student ID:1a)
	Development of nursing informatics competence	Q68. *Clinical nurses play an important role in the development of nursing informatics. First, as learners, they must have knowledge of nursing informatics, basic computer skills, and relevant software and information management skills. Then, as practitioners, they must apply the appropriate knowledge and awareness in their practical work.(Student ID:3b)*

Note. This table presents representative quotations supporting the themes and subthemes identified through qualitative analysis of students' perceptions of nursing informatics and digital health. The quotations illustrate the coding process and provide evidence for the interpretation of qualitative findings.
